# Ethanol extract of *Gynura bicolour* reduces atherosclerosis risk by enhancing antioxidant capacity and reducing adhesion molecule levels

**DOI:** 10.1080/13880209.2021.1912116

**Published:** 2021-04-27

**Authors:** Shu-Ling Hsieh, Jinn-Chyi Wang, Yun-Shan Huang, Chih-Chung Wu

**Affiliations:** a Department of Seafood Science, National Kaohsiung University of Science and Technology, Kaohsiung, Taiwan; b Department of Food Science and Technology, Tajen University, Pingtung, Taiwan; c Department of Food and Nutrition, Providence University, Taichung, Taiwan

**Keywords:** Antioxidant capacity, adhesion molecule, vascular endothelium

## Abstract

**Context:**

*Gynura bicolour* (Roxb. and Willd.) DC (Asteraceae) leaf is a common vegetable. Ethanol extracts of fresh *G. bicolour* leaves (GBEE) have several physiological effects, but studies on atherosclerosis are limited.

**Objective:**

We investigated the oxidant scavenging ability and vascular adhesion molecule expression of these extracts.

**Materials and methods:**

The antioxidant effects of 0.05–0.4 mg/mL GBEE were analyzed *in vitro*. Intracellular antioxidant capacity and adhesion molecule levels were detected in EA.hy926 cells pre-treated with 10–100 μg/mL GBEE for 8 h, then TNF-α for 3 h. The antioxidant capacity of red blood cells and the adhesion molecule levels in the thoracic aorta were detected in high-fat diet (HFD)-fed Sprague–Dawley rats treated with GBEE for 12 weeks.

**Results:**

The *in vitro* EC_50_ values of GBEE based on its DPPH radical-scavenging ability, reducing power, and ferrous ion-chelating ability were 0.20, 3.21 and 0.49 mg/mL, respectively. In TNF-α-treated EA.hy926 cells, the thiobarbituric acid-reactive substance levels were decreased after 10, 50, or 100 μg/mL GBEE treatments (IC_50_: 19.1 mg/mL). When HFD-fed rats were co-treated with GBEE, the GBEE-H group exhibited 25% higher glutathione levels than the HFD group (*p* < 0.05). E-selectin, intercellular adhesion molecule-1, and vascular cell adhesion protein-1 levels were decreased in TNF-α-treated EA.hy926 cells after GBEE treatment (by approximately 11–73%; *p* < 0.05), and the above three adhesion molecules levels were decreased in HFD-fed rats with combined GBEE treatment (by approximately 30–77%; *p* < 0.05).

**Conclusions:**

GBEE can protect the vascular endothelium by reducing adhesion molecule expression and regulating antioxidants. It may have the potential to prevent atherosclerosis.

## Introduction

*Gynura bicolour* (Roxb. and Willd.) DC (Asteraceae) is a common vegetable and traditional functional food in Taiwan and the Far East. The fresh leaves of *G. bicolour* are dark-green and purple on the top and bottom sides, respectively. There are many plant pigments and phytochemicals, including chlorophyll, gallic acid, β-carotene, rutin, anthocyanidin, myricetin, and morin (Wu et al. 2013, 2015). Chen et al. (2012) reported high contents of sesquiterpene compounds such as β-caryophyllene, α-caryophyllene, and α-copaene in *G. bicolour* (Chen et al. 2012). Previous literature has reported the antioxidant activity of the pigments and flavonoids of leafy vegetables such as chlorophyll A (Sarker and Oba 2019a), chlorophyll B (Sarker, Hossain and Oba 2020), betacyanins (Sarker and Oba 2020a), betaxanthins (Sarker, Oba, et al. 2020), carotenoids (Sarker and Oba 2020b), betalains (Sarker and Oba 2020c), phenolics (Sarker and Oba 2018a), flavonoids (Sarker and Oba 2020d), phenolic acids (Sarker and Oba 2018b), β-carotene (Sarker, Hossain, Iqbal, et al. 2020), rutin (Sarker and Oba 2020e), and myricetin (Sarker and Oba 2019b). Previous literature reported the antioxidant activity of pigments, and flavonoids of leafy vegetables such as flavanols (Sarker and Oba 2020f) and flavanones (Sarker and Oba 2020g). Lu et al. (2010) showed that the above components not only provide *G. bicolour* with its pigmentation but also may have physiologic effects. Previous studies have shown that water extracts of *G. bicolour* have anti-inflammatory (Wu et al. 2013) and antioxidant effects (Krishnan et al. 2015), promote iron bioavailability (Wu et al. 2015), exert anticancer (Teoh et al. 2016) and hepatoprotective effects (Yin et al. 2017), promote hypoglycaemia (Pai et al. 2019), protective of skin’s photodamage (Li et al. 2020), and decrease serum cholesterol levels (Hsieh et al. 2020). Because *G. bicolour* has antioxidant, anti-inflammatory, and serum cholesterol-lowering effects, the potential of *G. bicolour* to prevent atherosclerosis is worthy of investigation.

Atherosclerosis is one of the more common signs of cardiovascular disease. Pathological studies have shown that a series of changes, including the accumulation of oxidatively modified low-density lipoprotein (LDL) in the intima, which contributes to early-stage monocyte recruitment and transmigration, foam cell formation, and fibrous plaque formation in end-stage atherosclerosis, occurs in vessels during atherogenesis (Lara-Guzmán et al. 2018). Native LDL is not taken up by macrophages and does not form foam cells. Under oxidative stress, a large amount of oxidative LDL accumulates in the circulatory system, specifically in the blood vasculature, and rolls onto the vascular endothelium surface (Lara-Guzmán et al. 2018). The accumulation of oxidatively modified LDL and the inflammation of the intimal endothelium significantly contribute to monocyte recruitment and foam cell formation (Čejková et al. 2016). In addition, the accumulation of oxidized LDL stimulates endothelial cells to secrete proinflammatory molecules and adhesion molecules (Lara-Guzmán et al. 2018). Previous studies have shown that the consumption of a high-fat diet (HFD) and lipid peroxidation lead to an inflammatory response that triggers the production of reactive oxygen species (ROS; Du et al. 2012). Leukocyte and endothelial adhesion molecules, such as E-selectin, intercellular adhesion molecule-1 (ICAM-1), and vascular cell adhesion molecule-1 (VCAM-1), are also formed under inflammatory and oxidative stress conditions (Blankenberg et al. 2003). If the inflammatory response is reduced and the vascular endothelium remains intact, it may be possible to prevent endothelial dysfunction from progressing to atherosclerosis. In clinical therapy, for example, aspirin, probucol and HMG-CoA reductase inhibitors can protect against atherosclerosis by reducing the inflammatory response and adhesion molecule formation in the circulatory system (Chen et al. 2006). Therefore, reducing inflammation with dietary foods, thereby decreasing the need for medications that reduce chronic inflammatory responses, such as aspirin and indomethacin, is a good countermeasure to prevent atherosclerosis.

Oxidative stress-induced endothelial dysfunction is the other primary pathophysiologic mechanism of atherosclerosis and cardiovascular disease. Oxidative stress is involved in inflammation induction, enhanced adhesion molecule expression, and vascular endothelial layer damage (Lara-Guzmán et al. 2018). ROS can modulate cellular function and the overexpression of inflammatory cytokines and adhesion molecules (ICAM-1, VCAM-1, and E-selectin; Urso and Caimi 2011). Oxidant-mediated LDL oxidation and vascular injury are also crucial in atherogenesis (Yang et al. 2017). By contrast, the endogenous antioxidant ability of vascular tissues, which contain factors with antioxidant capabilities and include vitamin E, vitamin C, and glutathione, seems relevant to the prevention of atherosclerosis (Malekmohammad et al. 2019). Kevil et al. (2004) showed that as the level of GSH increases, the level of constitutive ICAM-1 decreases, and vice versa. There is a close relationship between redox signalling and inflammatory responses under the inflammation endothelium.

The objective of this study was therefore to investigate the effects of ethanol extracts of fresh *G. bicolour* leaves (GBEE) on antioxidant capacity and adhesion molecule protein expression in *in vitro* and *in vivo* models to determine the potential of *G. bicolour* to reduce atherosclerosis formation.

## Materials and methods

### Preparation of GBEE

In this study, *G. bicolour* plants, excluding the roots, were purchased from agricultural production and marketing groups in Yuanshan Village (Ilan, Taiwan) in August 2010. A voucher specimen plant was grown by the Department of Forestry, National Chung Hsing University, and identified by Yen Hsueh Tseng, Ph.D. A voucher specimen (TCF13549) has been deposited at National Chung Hsieh University (NCHU, Taichung, Taiwan).

GBEE was prepared as described in our previous study with modifications (Wu et al. 2015). The fresh leaves of the *G. bicolour* homogenates were extracted with ethanol (v/v: 1/1) by stirring on a stirring plate, which enhances extraction efficiency, for 6 h at 4 °C, then dried in a rotary vacuum dryer. The concentrated product was dried in a freeze dryer at −43 °C. The percent yield of GBEE was 0.9% (w/w). The GBEE was kept at −20 °C until being used in the study.

### Cell culture, animal feeding and treatments

The EA.hy926 human umbilical vein endothelial cell line was purchased from the Bioresource Collection and Research Centre (Hsinchu, Taiwan). The cells were maintained in Dulbecco’s modified Eagle medium (DMEM; Gibco; Thermo Fisher Scientific, Inc., Waltham, MA, USA) supplemented with 2 mM l-glutamine, 100 units/mL penicillin, 100 μg/mL streptomycin, and 10% (v/v) heat-inactivated foetal bovine serum (FBS; Gibco; Thermo Fisher Scientific, Inc.) at 37 °C in a humidified atmosphere of 5% CO_2_. The cells were plated at a density of 5 × 10^5^ in 30 mm culture dishes and were incubated until reaching 90% confluence. The cells were pre-treated with 10, 50 or 100 μg/mL GBEE for 8 h and then stimulated with or without 10 ng/mL TNF-α (R&D Systems, Minneapolis, MN, USA) for 3 h. TNF-α was used in this study to induce oxidative stress and endothelial dysfunction (Chen et al. 2008), and the induction period was selected based on a preliminary study. The GBEE was diluted in 95% ethanol, and cells treated with 95% ethanol alone served as the control group. Cells treated with 10 ng/mL TNF-α alone served as a positive control group.

It is known that long-term feeding with a HFD can enhance oxidative stress in rats (Du et al. 2012). Four-week-old male Sprague–Dawley (SD) rats were purchased from the National Laboratory Animal Centre (Taipei, Taiwan). After 1 week of acclimation, the rats were randomly assigned to five groups (*n* = 6) based on weight and house under a 12 h light cycle. The rats were used in compliance with the Guide for the Care and Use of Laboratory Animals (National Research Council (US) Committee 2011), and all the animal care and experimental protocols performed in the present study were approved by the Institutional Animal Care and Use Committee of Fooyin University (Kaohsiung, Taiwan). The rats were given free access to water and an AIN-93G-based diet (normal diet, ND; 5% soybean oil) or an HFD (20% soybean oil plus 0.5% cholesterol) (Dyets, Inc., Bethlehem, PA, USA). The rats fed with HFD were subdivided into GBEE-L, GBEE-M, GBEE-H, and control groups and orally administered 4, 8, or 16 mg/kg body weight (BW) GBEE or 40 mg/kg BW lovastatin (LOVA; Sigma, St. Louis, MO, USA), a clinical drug for hyperlipidaemia (Zeller and Uvodich 1988), as a positive control. The doses of GBEE used in this study were translated from our cell study results. The GBEE and LOVA were dissolved in 95% ethanol and administered orally 4 times per week for 12 weeks. At the end of the treatment, the rats were fasted overnight and sacrificed with carbon dioxide. Blood was withdrawn from their hearts, and their serum was prepared for biochemical analysis. The thoracic aorta, liver, heart, spleen, and kidney were removed, weighed, and inspected prior to pathological examination. The thoracic aorta and liver tissues were fixed in 10% neutral buffered formalin. Sections of the paraffin-embedded tissue (5 μm) were stained with haematoxylin and eosin (H&E). A pathologic examination was performed by an expert pathologist who was blinded to the experimental conditions. The fresh thoracic aorta and livers were removed, minced, quickly frozen in liquid nitrogen and stored at −80 °C. Before the biochemical analysis, the thoracic aortic tissue and fresh liver tissue homogenates were prepared in four volumes of homogenization buffer (10 mmol/L potassium phosphate, 150 mmol/L potassium chloride, and 1 mM PMSF, pH 7.4).

### Analysis of lipid peroxidation and GSH levels in EA.hy926 cells and the red blood cells of HFD-fed SD rats

Lipid peroxidation was evaluated by measuring the thiobarbituric acid-reactive substances (TBARS) with a fluorescence spectrophotometer (Hitachi F4500, Tokyo, Japan) as described by Fraga et al. (1988). At the end of the experimental treatment, the reaction was stopped by removing the medium and washing it with cold phosphate-buffered saline (PBS, 3.2 mM Na_2_HPO_4_, 0.5 mM KH_2_PO_4_, 1.3 mM KCl, and 135 mM NaCl, pH 7.4). The cells were removed with a cell scraper with 100 μL of 20 mM phosphate buffer containing 0.5% Triton X-100 and 10 mM butylated hydroxytoluene (in ethanol) and centrifuged at 10,000 *g* at 4 °C. Using 100 μL of the upper cell suspension, 2 mL of 0.1 N HCl, 0.3 mL of 10% phosphotungstic acid, and 1 mL of 0.7% 2-thiobarbituric acid were added to the mixture. The resulting mixture was heated for 30 min in boiling water, and the thiobarbituric acid-reactive substances (TBARS) were extracted into 5 mL of *n*-butanol. Following centrifugation at 3500 *g* and 4 °C for 15 min, the fluorescence of the butanol layer was measured with a fluorescence microplate reader (Bio-Tek Instruments). The excitation and emission wavelengths were 515 nm and 555 nm, respectively, within a fluorescence microplate reader (Bio-Tek Instruments). The protein concentrations of the EA.hy926 cell samples and the supernatants of rat samples were determined using the method by Sarker et al. (2016). The acid-soluble GSH levels in the EA.hy926 cells and hepatocellular supernatants were determined by following the method described by Wu et al. (2001). To analyse the GSH in the EA.hy926 cells, the reaction was stopped by removing the medium and washing it with cold phosphate-buffered saline (PBS). One millilitre of 1 mol/L perchloric acid containing 2 mmol/L 1,10-phenanthroline was added to each culture plate. The plates were scraped and the contents were centrifuged at 10,000 *g* for 10 min. The 400 µL supernatant was mixed with 40 µL iodoacetic acid (100 mM in PBS) for 15 min. After neutralization by potassium hydrogen carbonate, the acid-soluble GSH in the hepatocellular supernatant was measured by HPLC (Wu et al. 2001).

To analyze the GSH in the hepatic tissue, the liver tissue homogenates were centrifuged at 10,000 *g* for 30 min at 4 °C. To 400 µL of tissue homogenate sample, 1 mL mol/L perchloric acid containing 2 mmol/L 1,10-phenanthroline was added and the mixture was centrifuged at 10,000 *g* for 10 min. The sample was mixed with 40 µL of iodoacetic acid (100 mM in PBS) for 15 min. After neutralization by potassium hydrogen carbonate, the acid-soluble GSH in the hepatocellular supernatant was measured by HPLC (Wu et al. 2001).

### Analysis of the adhesion molecule protein levels in EA.hy926 cells and the thoracic aortas of SD rats fed with HFD

After the EA.hy926 cells were pre-treated with various concentrations of GBEE and with or without TNF-α as described above, they were washed twice with cold PBS and harvested in 200 μL of lysis buffer containing 10 mM Tris-HCl, 5 mM EDTA, 0.2 mM phenylmethylsulfonyl fluoride, and 20 μg/mL aprotinin, pH 7.4. After the collected cells were lysed by sonication, the expression levels of the cellular proteins were determined by following the method described by Sarker et al. (2016). Thoracic aortic tissue homogenates were prepared in four volumes of homogenization buffer (10 mmol/L potassium phosphate, 150 mmol/L potassium chloride, and 1 mM PMSF, pH 7.4) and centrifuged at 10000 *g* for 30 min at 4 °C. The protein concentration of the supernatants was determined using the method described by Sarker et al. (2016).

A total of 10 to 20 μg of cellular protein from each EA.hy926 cell or thoracic aortic tissue sample was loaded onto a 10% sodium dodecyl sulphate polyacrylamide gel (Laemmli 1970). After electrophoresis, the proteins were transferred to polyvinylidene difluoride membranes (Towbin et al. 1979). The membranes were then incubated with an anti-E-selectin (1:1000 dilution; cat. no. cd62e, Abcam Inc., Cambridge, MA, USA), anti-ICAM-1 (1:1000 dilution; cat. no. ab2213, Abcam), anti-VCAM-1 (1:1000 dilution; cat. no. ab98954, Abcam), or anti-β-actin antibody (1:10,000 dilution; cat. no. sc-47778, Santa Cruz Biotechnology) at 37 °C for 1 h and subsequently incubated with peroxidase-conjugated secondary antibodies. The bands were visualized using hydrogen peroxide/tetrahydrochloride diaminobenzidine (DAB; Sigma, St. Louis, MO, USA) and quantified using a ChemiDoc™ MP Imaging System (Bio-Rad Laboratories Inc., Hercules, CA, USA).

### EA.hy926 cell viability assay

To assess the optimal experimental dose of GBEE in this study, the viability of EA.hy926 cells was determined by following the method described by Denizot and Lang (1986) after the cells were pre-treated with various concentrations of GBEE and with or without TNF-α as described above. The cells were incubated in DMEM containing 0.5 mg/mL thiazolyl blue formazan (MTT; Sigma, St. Louis, MO, USA) for an additional 3 h, and the optical density (OD) was measured at a wavelength of 570 nm with an ELISA reader (Microplate Biokinetics reader, Bio-Tek Instruments, Winooski, VT, USA).

### *In vitro* antioxidant assays

The antioxidant effect of GBEE was investigated *in vitro* by measuring the DPPH (Sigma, St. Louis, MO, USA) radical-scavenging activity in 1 mL of a sample solution containing various concentrations of GBEE (0, 1, 2, and 4 mg/mL) according to the method described by Shimada et al. (1992). Vitamin C (1 mg/mL) was used as a control. The scavenging activity was calculated as (1 − A_GBEE_ or A_vitamin C_/A_blank_) × 100. The data are presented as percentages relative to the control (vitamin C). The reducing power was measured in 0.5 mL of a sample solution containing 0, 1, 2 or 4 mg/mL GBEE mixed with the reaction mixture (Shimada et al. 1992). Vitamin C (1 mg/mL) was used as a control. The reducing power was calculated as (A_GBEE_ or A_vitamin C_–A_blank_)/A_vitamin C_ × 100. The data are presented as percentages relative to the control (vitamin C). The chelation of ferrous ions by GBEE (0.5, 1, 2 or 4 mg/mL) was determined based on the method described by Dinis et al. (1994). The chelating activity was calculated as (A_GBEE_/A_EDTA_) × 100. The data are presented as percentages relative to the control (EDTA).

The superoxide radical-scavenging ability of 0.5, 1, 2, and 4 mg/mL GBEE was measured with commercial RANSOD kits (Randox Laboratories Ltd., San Diego, CA, USA) according to the manufacturer’s instructions.

### Statistical analysis

The data were analyzed using SPSS statistical analysis software for Windows, version 20.0 (SPSS Inc., Chicago, IL, USA). A one-way analysis of variance (ANOVA) and Duncan’s multiple-range test was used to evaluate the significance of differences between two mean values; *p*-values <0.05 were considered to indicate statistically significant results. The different letters (a, b, and c) indicate that a group is significantly different from each other group (*p* < 0.05).

## Results

### GBEE maintains the viability of EA.hy926 cells under TNF-α treatment and protects SD rats from organ damage induced by HFD

In our preliminary experiment, the viability of the EA.hy926 cells treated with 10, 50, 100, 500 or 1000 μg/mL GBEE was not significantly different from that of the control cells (data not shown). For reasons related to yield and solubility, we used 10, 50, and 100 μg/mL GBEE as the experimental doses in this study. The viability of the EA.hy926 cells was also not significantly different between the groups treated with various concentrations of GBEE (approximately 91–101%, data not shown) or the control group (100%). But, the cell viability of the TNF-α only group (83%) was significantly lower than the control group (*p* < 0.05). For morphological examination by inverted microscopy, the cell number and morphology were not significantly different between any GBEE-treated group and the control group. Thus, treating with 10, 50 or 100 μg/mL GBEE did not affect the viability of the EA.hy926 cells treated with TNF-α.

After 12 weeks of feeding, there were no significant changes in BW gain or average food intake between the HFD, LOVA, GBEE-L, GBEE-M, and GBEE-H groups (Table 1). The liver, heart, spleen, and kidney-related weights were not different between the ND, HFD, LOVA, GBEE-L, GBEE-M, and GBEE-H groups (Table 1). Additionally, the appearance of each of these organs was not different between each group (data not shown).

**Table 1.** Effects of GBEE on the growth characteristics and organ-related body weights of SD rats fed a HFD for 12 weeks.

	BW gain	Food intake	Heart	Liver	Kidney	Spleen
	g	g/day	% of BW			
ND	331 ± 33^b^	25.67	0.30 ± 0.03	3.17 ± 0.61	0.72 ± 0.05	0.17 ± 0.03
HFD	436 ± 71^a^	20.43	0.28 ± 0.05	2.93 ± 0.23	0.65 ± 0.07	0.15 ± 0.02
LOVA	448 ± 56^a^	21.38	0.26 ± 0.03	3.14 ± 0.38	0.61 ± 0.07	0.13 ± 0.01
GBEE-L	427 ± 66^a,b^	21.81	0.27 ± 0.01	3.12 ± 0.28	0.66 ± 0.04	0.14 ± 0.02
GBEE-M	440 ± 20^a^	21.93	0.28 ± 0.03	2.97 ± 0.39	0.65 ± 0.07	0.15 ± 0.04
GBEE-H	468 ± 83^a^	21.55	0.27 ± 0.02	2.90 ± 0.17	0.63 ± 0.07	0.13 ± 0.01

SD rats fed a HFD were subdivided into the GBEE-L, GBEE-M, GBEE-H, and control groups and orally administered 4, 8 or 16 mg/kg BW GBEE or 40 mg/kg BW LOVA as a positive control, respectively. The data are expressed as the mean ± SD of three independent experiments.

^a,b^Values of groups in the same cell phase marked with different letters differed significantly, as determined by Duncan’s test (*p* < 0.05).

**Figure 1.**
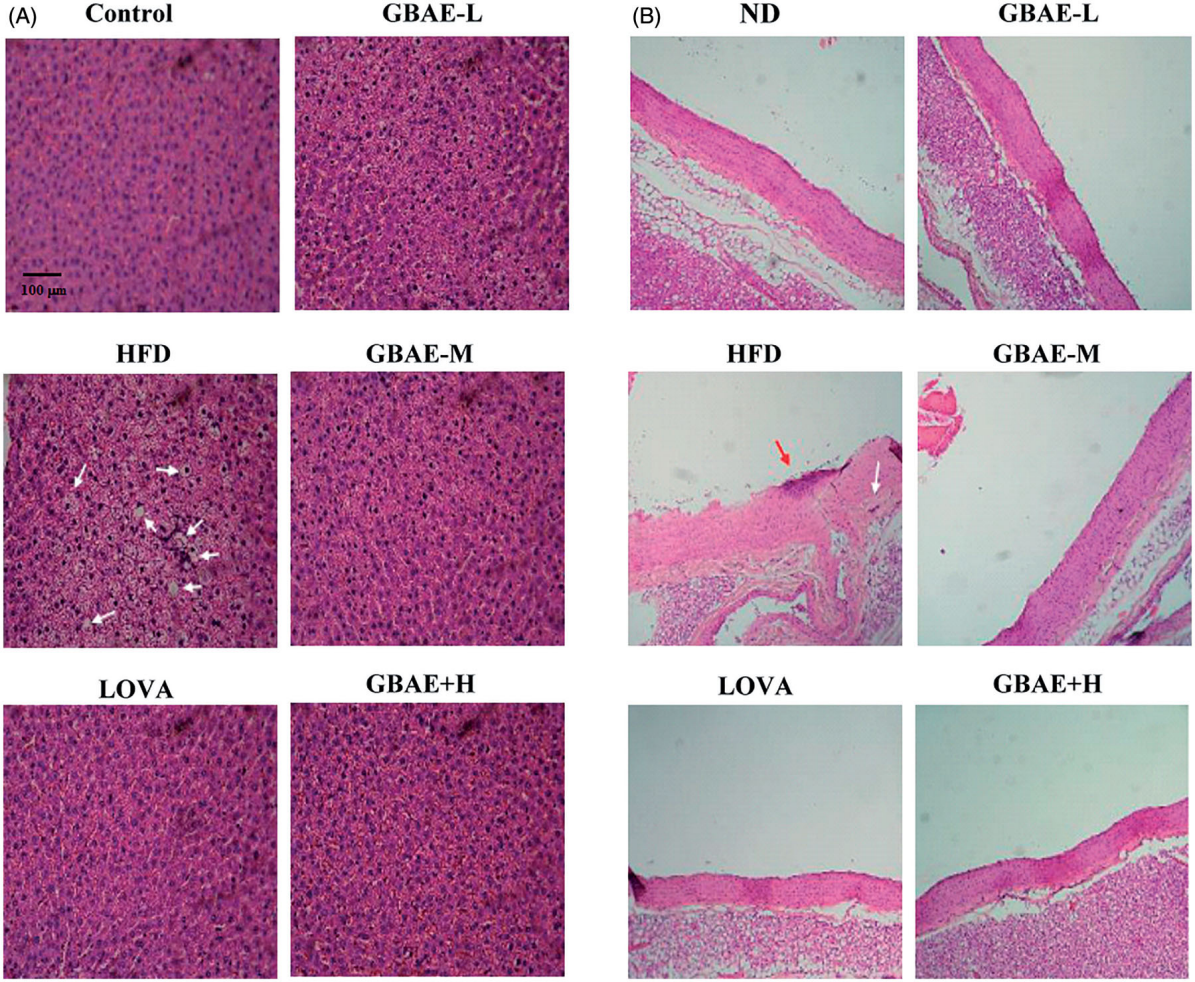
Pathologic examination of the livers and thoracic aortas of SD rats treated with GBEE for 12 weeks. SD rats consuming a HFD were subdivided into GBEE-L, GBEE-M, and GBEE-H, and control groups and orally administered 4, 8, or 16 mg/kg BW GBEE or 40 mg/kg BW LOVA as a positive control, respectively. After H&E staining, pathologic examinations of the hepatic tissues (A) and the thoracic aorta (B) were performed. Fatty liver changes, steatosis, and endothelial inflammation are indicated by arrows.

In the animal experiments, a histologic examination was performed to detect fatty liver changes and thoracic aorta integrity. Feeding SD rats an HFD for 12 weeks led to fatty liver (Figure 1(A)). When co-treated with LOVA, GBEE-L, GBEE-M, or GBEE-H, the fatty liver changes were suppressed. In the ND group, the intima of the aorta was smooth, and the endothelial cells in the aorta were intact (Figure 1(B)). In the HFD group, the endothelial cells in the aorta were damaged, as shown by the dark red region in Figure 1(B), the intima of the aorta was thickened, the middle membrane was thin, and the inner membrane was covered with cells. However, significant improvements in the thickening of the endothelial cells, the intima, and the middle membrane were observed in the LOVA group and all of the GBEE treatment groups.

### GBEE decreases lipid peroxidation and enhances glutathione levels in EA.hy926 cells and SD rats

As shown in Table 2, the EA.hy926 cells in which oxidative stress was induced by TNF-α (0.48 ± 0.02 nmol/mg protein), the TBARS level was significantly higher than that of the control group (0.20 ± 0.01 nmol/mg protein; *p* < 0.05). In cells treated with TNF-α or one of the three concentrations of GBEE, the TBARS level was significantly decreased by approximately 25–43% relative to the level in the TNF-α group (IC50: 19.1 µg/mL; *p* < 0.05). In addition, the intracellular GSH level in TNF-α-induced cells treated with 100 µg/mL GBEE (53.4 ± 5.1 nmol/mg protein) was significantly higher than that of the TNF-α group (45.2 ± 3.6 nmol/mg protein; EC50: 11.03 nmol/mg protein; *p* < 0.05; Table 2). These results suggested that GBEE may reduce lipid peroxidation through increased intracellular GSH levels.

**Table 2.** Effects of GBEE on TBARS and GSH levels in EA.hy926 cells.

	TBARS	GSH
	nmol/mg protein	
Control	0.20 ± 0.01^b^	51.6 ± 2.6^a^
TNF-α (10 ng/mL)	0.48 ± 0.02^a^	45.2 ± 3.6^b^
TNF-α + GBEE (µg/mL)		
10	0.36 ± 0.02^b^	46.3 ± 2.7^a,b^
50	0.29 ± 0.02^b^	48.2 ± 2.4^a,b^
100	0.27 ± 0.01^b^	53.4 ± 5.1^a^
GBEE (100 μg/mL)	0.19 ± 0.01^b^	58.0 ± 4.2^a^

EA.hy926 cells were pre-treated with 10, 50 or 100 μg/mL GBEE for 8 h and then stimulated with or without 10 ng/mL TNF-α for 3 h. EA.hy926 cells treated with alcohol served as a control group. The data are expressed as the mean ± SD of three independent experiments.

^a,b^Values of groups in the same cell phase marked with different letters differed significantly, as determined by Duncan’s test (*p* < 0.05).

The red blood cell TBARS levels of the HFD group rats were significantly higher than those of the ND group rats after 12 weeks of HFD feeding (0.18 ± 0.02 nmol/mg protein; *p* < 0.05; Table 3). In the three GBEE groups, the red blood cell TBARS levels were significantly lower than that of the control group after 12 weeks of HFD feeding (0.07–0.09 mmol/mg protein; *p* < 0.05). In addition, the red blood cell GSH level was significantly higher in the GBEE-M and GBEE-H groups than in the HFD group (*p* < 0.05; Table 3). These results show that GBEE can improve the blood antioxidant status, that is, enhance red blood cell GSH levels, leading to reduced lipid peroxidation, that is, decreased TBARS levels, in SD rats consuming an HFD.

**Table 3.** Effects of GBEE on TBARS and GSH levels in the red blood cells of SD rats fed a HFD for 12 weeks.

	TBARS	GSH
	nmol/mg protein	
ND	0.07 ± 0.01^b^	0.36 ± 002^a^
HFD	0.18 ± 0.02^a^	0.25 ± 0.02^b^
LOVA	0.11 ± 0.02^b^	0.30 ± 0.05^a,b^
GBEE-L	0.09 ± 0.02^b^	0.32 ± 0.04^a,b^
GBEE-M	0.07 ± 0.01^b^	0.35 ± 0.01^a^
GBEE-H	0.09 ± 0.01^b^	0.38 ± 0.02^a^

SD rats fed a HFD were subdivided into the GBEE-L, GBEE-M, GBEE-H, and control groups and orally administered 4, 8 or 16 mg/kg BW GBEE or 40 mg/kg BW LOVA as a positive control, respectively. The data are expressed as the mean ± SD of three independent experiments.

^a,b^Values of groups in the same cell phase marked with different letters differed significantly, as determined by Duncan’s test (*p* < 0.05).

### GBEE reduces adhesion molecule protein levels in EA.hy926 cells and the thoracic aortas of SD rats given a HFD

**Figure 2.**
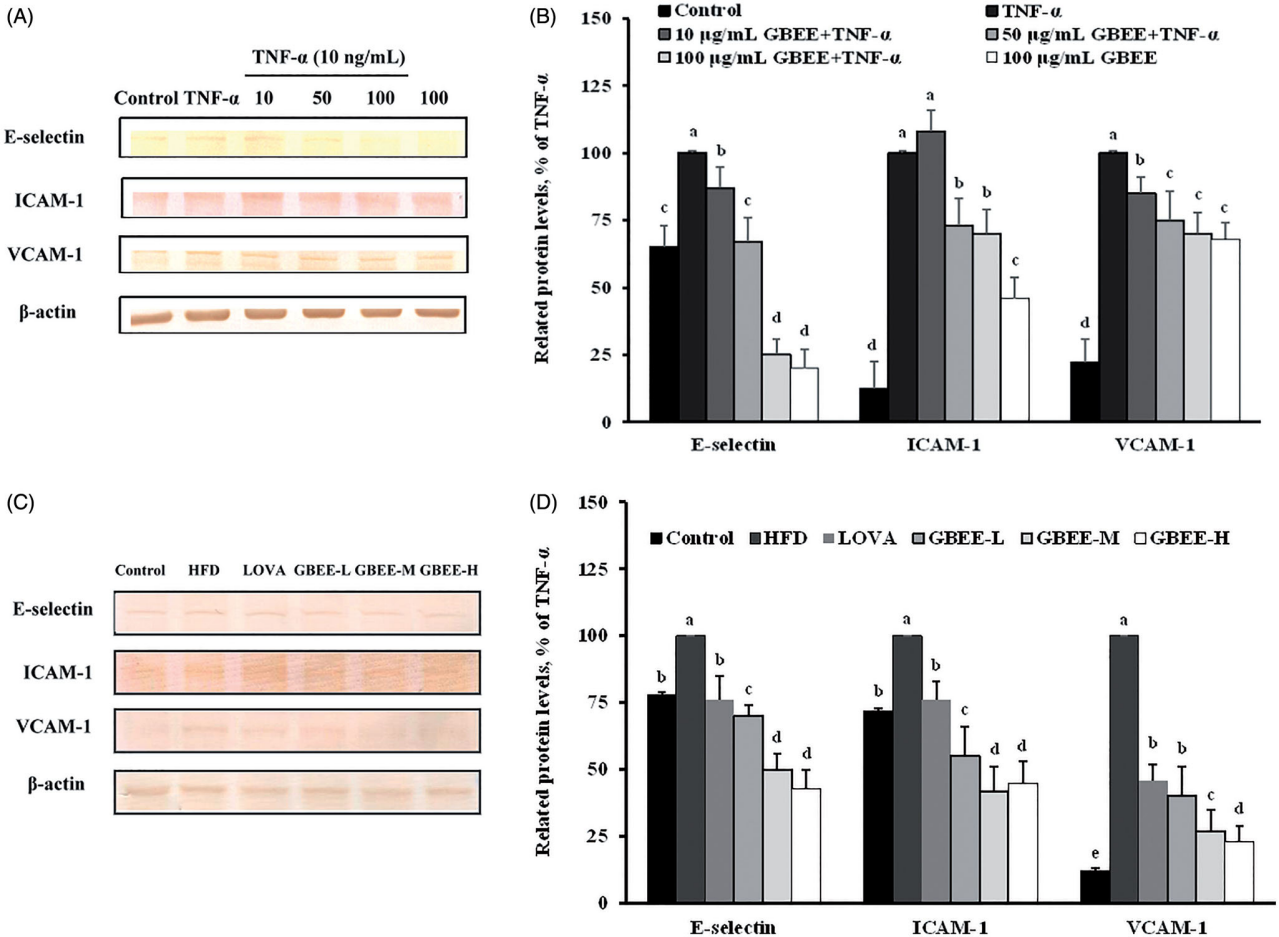
Effect of GBEE on the protein levels of E-selectin, ICAM-1, and VCAM-1 in EA.hy926 cells and SD rats. EA.hy926 cells were pre-treated with 10, 50 or 100 μg/mL GBEE for 8 h and then stimulated with or without 10 ng/mL TNF-α for 3 h. EA.hy926 cells treated with alcohol served as the control group. SD rats receiving a HFD were subdivided into the GBEE-L, GBEE-M, and GBEE-H, and control groups and orally administered 4, 8 or 16 mg/kg BW GBEE or 40 mg/kg BW LOVA as a positive control, respectively. Immunoblot assays were performed to analyze the expression levels of E-selectin, ICAM-1, and VCAM-1 in the EA.hy926 cells (A) and the thoracic aorta (C). Quantifications of the data are shown in (B) and (D). The data are expressed as the means ± SDs of three independent experiments. ^ab^The values are significantly different those of the other groups, as determined by Duncan’s test (*p* < 0.05).

The E-selectin, ICAM-1, and VCAM-1 protein levels were significantly higher in EA.hy926 cells treated with TNF-α for 24 h than in the control cells (*p* < 0.05; Figure 2(A,B)). When the cells were pre-treated with 10, 50 or 100 μg/mL GBEE for 8 h and then stimulated with or without 10 ng/mL TNF-α, the E-selectin, ICAM-1 and VCAM-1 protein levels were significantly decreased by 11–3%, 25–32% and 12–28%, respectively, compared with the TNF α-treated control group (*p* < 0.05; Figure 2(B)).

The protein levels of E-selectin, ICAM-1, and VCAM-1 in the thoracic aorta were significantly higher in the HFD-rats than in the ND-fed control rats (*p* < 0.05; Figure 2(C,D)). The E-selectin, ICAM-1, and VCAM-1 protein levels in the thoracic aorta were significantly lower in SD rats co-administered an HFD and LOVA or any of the three doses of GBEE than in the HFD-fed rats (*p* < 0.05). In SD rats treated with GBEE-L, GBEE-M, or GBEE-H and fed an HFD, the E-selectin, ICAM-1 and VCAM-1 protein levels were significantly decreased by 32–57%, 25–67% and 56–89%, respectively, compared with those in the HFD group (100%; *p* < 0.05; Figure 2(D)).

### GBEE has antioxidant effects *in vitro*

**Figure 3.**
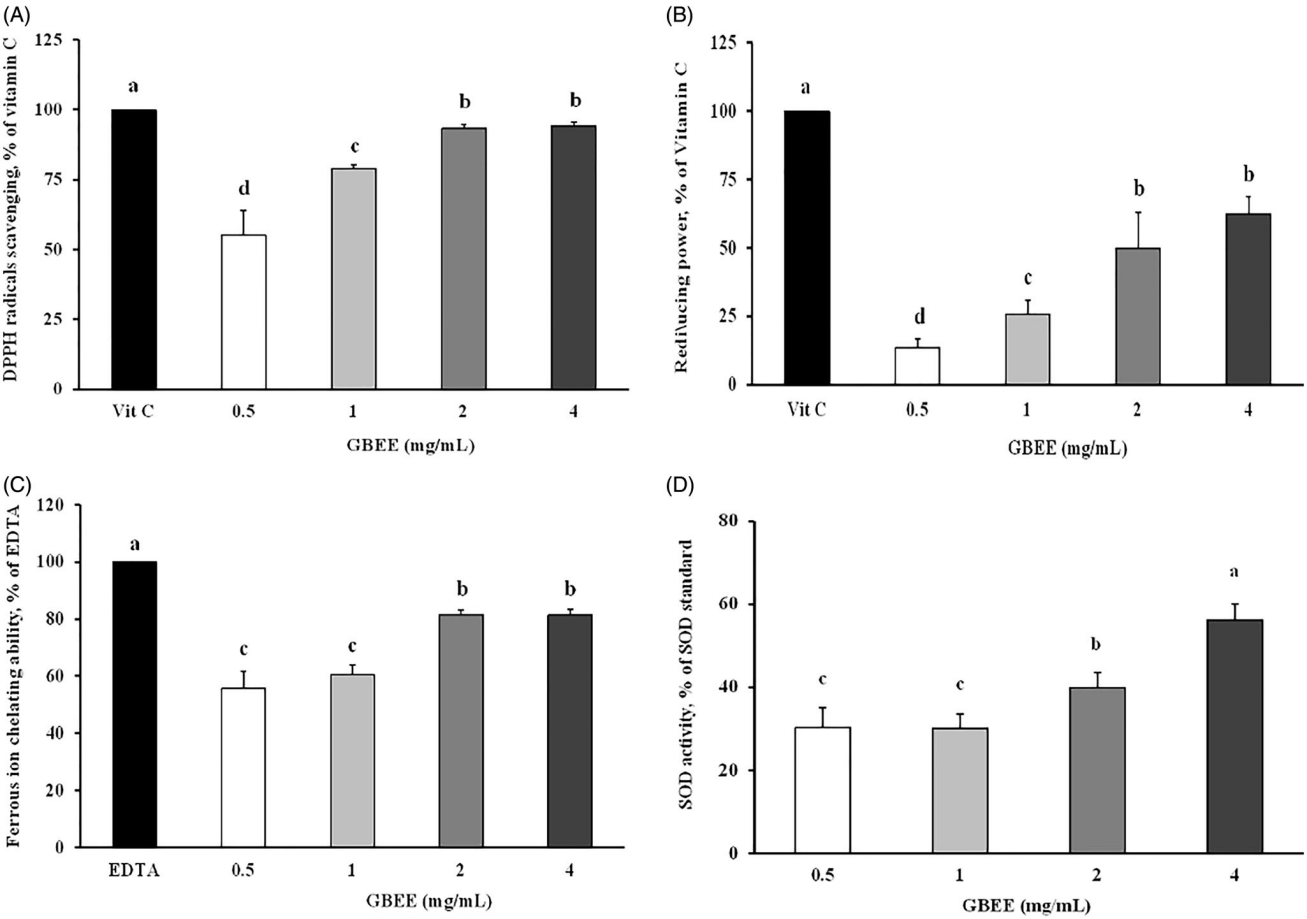
Analysis of the antioxidant activity of GBEE *in vitro*. (A) DPPH radical scavenging activity. (B) Reducing power. (C) Ferrous ion-chelating ability. (D) Superoxide radical-scavenging activity. The data are expressed as the means ± SDs of three individual experiments. ^abcd^Values are significantly different from those of the other groups, as determined by Duncan’s test (*p* < 0.05). *p*-Values are <0.05 compared to the vitamin C, Na_2_EDTA, or SOD standard groups.

In the present study, the DPPH-mediated reduction of free radicals observed in the 0.5, 1, 2 and 4 mg/mL GBEE-treated groups was 55.0 ± 8.9%, 79.0 ± 1.8%, 93.2 ± 1.4% and 94.1 ± 1.4% of that observed in the vitamin C-treated group (100%), respectively (Figure 3(A)). The reducing power of 0.5, 1, 2 and 4 mg/mL GBEE reached 13.4–62.3% of that of vitamin C (100%; Figure 3(B)). Figure 3(C) shows that the ferrous ion-chelating abilities of the 0.5, 1, 2, and 4 mg/mL GBEE-treated groups were 55.6 ± 6.0%, 60.5 ± 3.5%, 81.5 ± 1.5% and 81.4 ± 2.0% of those of EDTA (100%; Figure 3(C)), respectively. The superoxide radical-scavenging ability of GBEE was 30–56% of that of the superoxide dismutase (SOD) standard (Figure 3(D)). These results showed that the EC_50_ values of GBEE based on its DPPH radical-scavenging ability, reducing power, and ferrous ion-chelation ability were 0.20, 3.21 and 0.49 mg/mL, respectively.

## Discussion

We showed that GBEE significantly inhibited the protein expression of blood adhesion molecules, including E-selectin, ICAM-1, and VCAM-1, in human endothelial cells treated with TNF-α and in SD rats fed with an HFD. Additionally, GBEE alleviated the damage to the thoracic aortas and hepatic tissues of SD rats that consumed an HFD. These findings suggest that the inhibition of adhesion molecule expression by GBEE may be a result of its antioxidant ability. These novel findings showed that *G. bicolour* has the potential to suppress atherosclerosis by reducing adhesion molecule activity.

Atherosclerosis is known to result from the inflammatory response and oxidative stress. Oxidative stress is caused by inflammatory cytokine accumulation (Lusis 2000). Mukherjee et al. (2007) showed that TNF-α secretion increased ROS production and depleted the levels of intracellular GSH. An increase in the ROS concentration in endothelial cells causes vascular tissue damage and remodelling (Bretón-Romero and Lamas 2014). NF-κB signalling is activated in this pro-oxidant state, leading to the induction of adhesion molecule expression and the promotion of these functions of adhesion molecules along the endothelial cell surface (Ju et al. 2010). The expression of vascular adhesion molecules, such as E-selectin, ICAM-1, and VCAM-1, plays an important role in endothelial cell dysfunction and atherosclerosis (Blankenberg et al. 2003). Therefore, enhancing the antioxidant ability of the vascular system is an important method for preventing atherosclerosis. In this study, GBEE decreased the TBARS levels by increasing the GSH levels. Raising the GSH levels may protect against damage from oxidative stress induced by TNF-α or HFD. The antioxidant ability of GBEE was evidenced by an increase in antioxidant capacity in the *in vitro* model and enhanced GSH production in the cell culture model as well as by increased GSH levels in the animal model.

Our previous study (Hsieh et al. 2013) showed that GBEE was rich in flavonoids and chlorophyll. These flavonoids and chlorophyll may give GBEE its antioxidant potential to regulate adhesion molecule expression. Previous studies (Bhaskar et al. 2016) have also shown that flavonoid- and polyphenol-rich materials can significantly inhibit the effects of adhesion molecules. For example, quercetin can reduce ICAM-1 and VCAM-1 levels by decreasing ROS levels and increasing GSH levels in human umbilical vein endothelial cells. β-Sitosterol, a polyphenolic compound in the genus *Piper* (Piperaceae), can significantly inhibit TNF-α-induced ICAM-1, VCAM-1, and E-selectin expression in HUVECs (Gupta et al. 2010). Purple sweet potato leaf extract and its polyphenolic components can decrease the adhesion ability and ICAM-1 and VCAM-1 expression in human umbilical vein endothelial cells treated with TNF-α (Chao et al. 2013). The above materials and compounds may be rich in flavonoids, a polyphenolic group that has a strong ability to scavenge ROS to prevent immune responses and oxidative stress-induced damage and reduce adhesion molecule levels. It is worth noting that some non-flavonoids or non-polyphenolic compounds can also decrease adhesion molecule expression through increasing antioxidant ability. For example, vitamin D supplementation can decrease ROS and ICAM-1 levels and increase GSH levels in human umbilical vein endothelial cells treated with ketone acetoacetate (Kanikarla-Marie and Jain 2016). Sulforaphane, a phytochemical derived from cruciferous vegetables, reduces adhesion molecule expression in human umbilical vein endothelial cells treated with LPS (Cho et al. 2016). An oligomeric proanthocyanidin derived from *Rhodiola rosea* L. (Crassulaceae) was shown to have anti-atherosclerosis effects in Wistar rats receiving a HFD, in which the superoxide dismutase and glutathione peroxidase activities are increased and the malondialdehyde, ICAM-1, and VCAM-1 levels are decreased (Zhou et al. 2018). The above studies provide evidence that decreases in the ICAM-1 and VCAM-1 levels due to an increase in antioxidant ability is an important means for preventing atherosclerosis. The ability of GBEE to inhibit atherosclerosis may be associated with its rich content of polyphenols and strong potential for enhancing antioxidant ability in various experimental models to decrease adhesion molecule expression. However, the mechanism by which GBEE inhibits adhesion molecule expression requires more study.

Leukocyte adhesion molecules can directly contribute to inflammatory responses within the blood vessel wall by increasing endothelial cell activation and augmenting the formation of atherosclerotic plaques (Lawson and Wolf 2009). Dysfunction in the endothelial lining of lesion-prone areas of the arterial vasculature is an important pathologic marker of atherosclerosis (Gimbrone and García-Cardeña 2016). Feeding SD rats an HFD can induce fatty liver and vascular intimal injury by increasing inflammation and oxidative stress (Libby et al. 2009; Yang et al. 2017). In this study, GBEE significantly decreased small fat-filled vacuoles in the cytosol of liver tissues of SD rats receiving an HFD, thus preventing liver damage. In addition, GBEE significantly alleviated the damage to the endothelial layer of the rat thoracic aorta induced by inflammatory and oxidative stress, including the thickening of the intima, the thinning of the middle membrane, and the covering of the inner membrane with cells. Previously, Wu et al. (2013) showed that GBEE can reduce the levels of molecular nitric oxide (NO) and prostaglandin E_2_ (PGE_2_) during inflammation by inhibiting the activation of NF-κB signalling. This mechanism could underlie the anti-inflammatory effect of GBEE in preventing thoracic aorta endothelial damage. Additionally, the excellent antioxidant ability of GBEE may provide a protective effect against fatty liver and endothelial damage. A previous study (Zhu et al. 2015) showed that when mice with hyperlipidaemia (induced by treatment with a 1.25% cholesterol diet for 30 days) were treated with *Lycium barbarum* L. (Solanaceae) polysaccharides, damage to the thoracic aorta endothelium was significantly alleviated. In this case, a significant decrease in the levels of IL-6 and malondialdehyde, which are indicators of inflammation and oxidative stress, respectively, may be important factors in protecting the thoracic aorta endothelium from *L. barbarum* polysaccharides. In another study (Jiang et al. 2017), in SD rats administered an HFD (containing 1% cholesterol) for 6 weeks, an ethanol extract of black mulberry fruit reduced fatty liver and ameliorated damage to the intima and media membrane of the thoracic aorta endothelium by enhancing the antioxidant ability, including by increasing GSH levels and the activities of related enzymes. Oligomeric proanthocyanidins from *R. rosea* have also been shown to alleviate damage to the thoracic aorta endothelium significantly by increasing superoxide dismutase and glutathione peroxidase activities and decreasing malondialdehyde and IL-1β, IL-6 and TNF-α levels in Wistar rats receiving an HFD (containing 1% cholesterol and 10% lard; Zhou et al. 2018). These studies showed that the abilities of GBEE and other phytochemicals to reduce oxidative stress, inhibit inflammation and preserve the endothelial layer of the thoracic aorta are important for the anti-atherosclerotic actions of these compounds.

## Conclusions

GBEE can reduce adhesion reaction in the atherosclerosis process. Its mechanism involves an enhancement of the antioxidant ability by GBEE to reduce adhesion molecule expression and protect the thoracic aorta endothelium. These findings may provide important insights for the potential discovery and development of GBEE as a novel functional food; however, the regulation of the molecular mechanism requires further research.

## Data Availability

The datasets used and/or analysed during the present study are available from the corresponding author on reasonable request.
